# Racial and cultural minority experiences and perceptions of health care provision in a mid-western region

**DOI:** 10.1186/s12939-018-0744-x

**Published:** 2018-03-16

**Authors:** Stephane M. Shepherd, Cynthia Willis-Esqueda, Yin Paradies, Diane Sivasubramaniam, Juanita Sherwood, Teresa Brockie

**Affiliations:** 1 Department of Mental Health, Bloomberg School of Public Health, Johns Hopkins University; Centre for Forensic Behavioural Science, Swinburne University of Technology, Baltimore, USA; 2 Department of Psychology, University of Nebraska-Lincoln, Lincoln, USA; 3 Alfred Deakin Research Institute for Citizenship and Globalisation, Deakin University, Geelong, Australia; 4 School of Psychological Sciences, Swinburne University of Technology, Hawthorn, Australia; 5 National Centre for Cultural Competence, University of Sydney, Camperdown, Australia; 6 School of Nursing, Johns Hopkins University, Baltimore, USA

**Keywords:** Health disparities, Cultural competence, Cultural matching, Minority health, Health care discrimination

## Abstract

**Background:**

Disparities across a number of health indicators between the general population and particular racial and cultural minority groups including African Americans, Native Americans and Latino/a Americans have been well documented. Some evidence suggests that particular groups may receive poorer standards of care due to biased beliefs or attitudes held by health professionals. Less research has been conducted in specifically non-urban areas with smaller minority populations.

**Methods:**

This study explored the self-reported health care experiences for 117 racial and cultural minority Americans residing in a Mid-Western jurisdiction. Prior health care experiences (including perceived discrimination), attitudes towards cultural competence and satisfaction with health care interactions were ascertained and compared across for four sub-groups (African-American, Native American, Latino/a American, Asian American). A series of multiple regression models then explored relationships between a concert of independent variables (cultural strength, prior experiences of discrimination, education level) and health care service preferences and outcomes.

**Results:**

Overall, racial/cultural minority groups (African Americans, Native Americans, Latino/a Americans, and Asian Americans) reported general satisfaction with current healthcare providers, low levels of both health care provider racism and poor treatment, high levels of cultural strength and good access to health care services. Native American participants however, reported more frequent episodes of poor treatment compared to other groups. Incidentally, poor treatment predicted lower levels of treatment satisfaction and racist experiences predicted being afraid of attending conventional health care services. Cultural strength predicted a preference for consulting a health care professional from the same cultural background.

**Conclusions:**

This study provided a rare insight into minority health care expectations and experiences in a region with comparatively lower proportions of racial and cultural minorities. Additionally, the study explored the impact of cultural strength on health care interactions and outcomes. While the bulk of the sample reported satisfaction with treatment, the notable minority of participants reporting poor treatment is still of some concern. Cultural strength did not appear to impact health care behaviours although it predicted a desire for cultural matching. Implications for culturally competent health care provision are discussed within.

## Background

Racial and cross-cultural health disparities in the United States are well documented [1–3]. Reports have identified gaps across a number of health indicators between the general population and particular cultural groups including African Americans, Native Americans and Latino/a Americans [3, 4]. For example, African Americans and Native Americans have lower life expectancies and higher infant mortality rates compared to the general population [4, 5]. Again, compared to the general population, African Americans have higher rates of mortality from heart disease and cancer [3]; Native Americans suffer higher rates of heart and liver disease [3, 5] and Latino/a Americans have higher rates of diabetes and liver disease [3, 4, 6]. The total prevalence of diabetes has also been found to be much higher among Asian Americans compared to White Americans [4]. Explanations for cross-cultural disparities in health include poverty, socio-economic status, discrimination, language barriers, lower health literacy and a lack of health service provider cultural competence [3].

In addressing the different levels at which racial or cross-cultural inequities may occur, Jones [7] and Cross et al. [8] point to: differential care within the health system, differential access to the health system, and differences in health-risk exposures and life opportunities. Differential care refers to biased beliefs or attitudes held and/or treatment decisions made by health professionals based on a patient’s race/culture. While racial classifications are often constructed on the basis of phenotypical distinctions, culture refers to aspects of life (i.e., norms, customs, beliefs, behaviours, social institutions) that an individual shares with others within a defined population. A body of evidence suggests that particular minority groups of color receive a poorer standard of health care [3, 9]. There is also evidence of both health care provider racism [10–12] and unconscious racial biases [13–15]. However, findings are mixed as to whether such bias directly underpins the reported lower quality of care and/or poorer health outcomes for particular minority groups [13, 15, 16]. While findings are still unclear in relation to practitioner bias and patient outcomes, studies indicate that particular minority groups are still more likely to hold negative perceptions of health care professionals and services [17–19]. This perhaps stems from prior experiences of discrimination with health services, differing health beliefs and expectations of care. Some racial/cultural groups for example subscribe to holistic conceptualisations of health [20–22]. The individualistic nature of western bio-medical health models and services may be viewed as limiting for those whose cultural health beliefs comprise spiritual and meta-physical components [23]. For Indigenous peoples in particular, both the imposition of western health systems and historical injustices committed within Western health institutions are believed to contribute to poor health and health service mistrust [24, 25]. The United States Commission on Civil Rights [26] found significant cultural barriers to health access and treatment for Native Americans, much of which is underpinned by the under-resourcing and structural inadequacies of the Indian Health Service.

The extant evidence appears to underscore several important conclusions regarding cross-cultural health care in the USA. First, health care provider discrimination exists. Second, those who report discrimination are more likely to report unmet clinical needs. Third, particular cultural groups disproportionately report poorer health interactions and outcomes. Four, such groups are more likely to hold negative perceptions of health care services. The relationships between these outcomes – healthcare provider racism, negative perceptions of services and patient outcomes – have received some attention in the literature. However, the effect of a patient’s cultural strength (the extent of an individual’s pride and connection to aspects of their culture) in relation to these outcomes has rarely been explored (cf [27]). This warrants academic attention for two reasons. First, it is predominantly minority groups who report discriminatory experiences in health care settings; and second, there is a growing body of evidence linking stronger cultural identity to positive health outcomes, including the mitigation of racism-induced distress [28–34].

This paper attempts to bridge these research findings by exploring the effect of cultural strength on minority health care experiences and outcomes. First, we aimed to ascertain levels of cultural strength, prior health care experiences (including perceived discrimination), attitudes towards cultural competence and satisfaction with health care interactions across a number of minority cultural groups in a Midwestern major city. Based on prior research, we expected African-American and Native-American participants to report higher levels of health care provider discrimination and lower levels of treatment satisfaction. We also anticipated that Native American participants would be more likely to report higher group levels of cultural strength. Our second aim explored the effect of cultural strength and prior experiences of health care discrimination on attitudes towards cultural competence and treatment satisfaction. We predicted that prior discrimination will be connected to lower treatment satisfaction and an aversion to accessing western health care services; and that a stronger connection to culture will be associated with support and a desire for culturally competent care.

## Method

### Participants

Participants were recruited at two sites in the capital city of a Midwestern state. The sites included a major shopping mall and the grounds of an annual cultural festival. The locations were chosen to increase access to minority populations. Inclusion criteria included; age 18 years or older, and self-identification as a member of a minority group (i.e., African American, Native American, Asian American, and Hispanic/Latino American). Almost 90% of the state’s population are white, 10.7% are Latino/a, 5.0% are African American, 2.5% Asian and 1.4% Native American [35]. As such, the four non-white groups above were oversampled in the study. There was a greater emphasis on oversampling Asian and Native American sub-groups in particular, given their smaller populations relative to Latino/a and African American populations. The age range (18 years and over) was selected as the study focuses on adults.

#### Demographic characteristics

Data was collected for 117 (Female = 70; Male = 47) participants. Almost three-quarters (*N* = 86) of the sample was collected from the shopping mall. The mean age of the sample was 31.94 (*SD*: 12.59) years. The self-identified cultural backgrounds of the participants included Latino/a American (*N* = 33, 28.2%), Native American (*N* = 32, 27.4%), African American/Black (*N* = 26, 22.2%), Asian American (*N* = 15, 12.8%) and Other (*N* = 11, 9.4%). All but one of the Native American sub-sample (*N* = 31, 97%) was collected at the cultural festival.

### Procedure

Data was collected across four days throughout September 2016. Data collection occurred on three days at the shopping mall and on one day at the annual cultural festival. The festival is held on one day in September annually. Researchers were stationed at stalls across both data collection sites. Research stalls displayed an advertising poster visible to members of the public. The poster included a brief statement promoting the study (i.e., you are invited to participate in our research study on perceptions of health care). The poster also encompassed the university logo and participant age restrictions. Prospective participants would then approach the stall for further information. To maximize sample numbers, our multi-cultural research term also actively approached prospective participants and directed them towards the stall. Minority individuals who were in proximity to the stall were approached by researchers where possible. Interested individuals over the age of 18 years who self-identified as a minority were then invited to have the study explained to them. If participants agreed to participate they were then asked to read (or have read to them) a consent form outlining the requirements and their involvement in the study. After consenting, participants then completed the anonymous online health experiences survey. Participants had the option of having researchers read the online questionnaire to them, if desired. The duration of participation ranged from 5 to 15 min. All participants received a $10 shopping voucher for their contribution. Bi-lingual researchers were available for Spanish-speaking participants.

### Measures

Participants completed a 14-item cross-cultural health service experiences questionnaire ascertaining their social, cultural, health needs and expectations as health service users, experiences of discrimination in health care settings, and how well existing health services accommodate their particular needs. Discrimination was gauged through perceived experiences of direct racism and poor treatment due to one’s cultural background. A perception of being treated poorly can refer to any experience of discrimination during a health care encounter beyond direct racism (i.e., poor communication, being misinformed, not provided with the whole range of treatment options, judgemental staff, feelings of not being respected, needs not met). A ‘cultural strength’ composite measure was created by averaging the items ‘Are you proud of your cultural heritage?’, ‘Do you feel connected to your culture?’ and ‘How important is your culture to your daily life?’ A consideration of these three components (pride, connection, importance) allowed for a more holistic appraisal of an individual’s cultural strength.

Participants were informed that the questions pertained to their experiences with conventional health services (not inclusive of traditional or cultural healing services). They were asked the extent to which they agree or disagree with various statements (i.e., “Do you think it is important for health care staff to know about different cultures?”; “Do you feel that you are treated poorly by healthcare professionals because of your cultural background?” (5 = Strongly Agree, 1 = Strongly Disagree). The item “How often do you visit health care services” was scored differently (1 = Never, 2 = Less than 5 times per year, 3 = 5–10 times per year, 4 = More than 10 times per year). Demographic information on race, gender and level of education was also obtained. The questionnaire was developed by the study authors after a consideration of public health, population health, and cultural competence literature bases and prior broad-based health surveys administered to Indigenous and cultural minority populations in North America, Australia and New Zealand. Questions were selected based on their relevance to the study and their alignment with cultural competence and cultural safety principles. No previous validated questionnaire specifically tailored to cultural minority health care experiences, cultural needs and strengths was available for use.

### Data analyses

Descriptive statistics were employed to characterise the sample (i.e., mean scores on questionnaire items by cultural group). An Analysis of variance (ANOVA) and subsequent post-hoc tests were conducted to ascertain cultural group differences across item scores. ANOVA analyses identifies if there are significant differences on mean item scores across groups. Bonferroni correction was applied and the adjusted significance value was set at < .01. Post-hoc tests confirm where significant differences occur between cultural groups. A series of multiple regression analyses were then employed to identify the influence of particular items (experiences of provider racism, perceptions of poor treatment, age, level of education, cultural strength) on a variety of different health provider experiences and outcomes (satisfaction with treatment, fear of visiting health care services, preference for a culturally-matched health service provider).

## Results

### Descriptive statistics

#### Overall sample

Mean scores and the prevalence of questionnaire items were first conducted for the total sample (*N* = 117) which was inclusive of the ‘other’ cultural category. The majority of the sample (*N* = 71, 60.7%) reported visiting health care services less than five times per year. Participants on average reported easy access to health services (*M* = 4.62, *SD* = .99).

On questions referring to cultural background, participants were generally very proud of their cultural heritage (*M* = 4.87, *SD* = .36), were often connected to their culture (*M* = 4.43, *SD* = .97), and felt that their culture was very important to their everyday life (*M* = 4.23, *SD* = 1.00). Questions pertaining to diversity in health care revealed that staff knowledge of different cultures was viewed as important (*M* = 4.56, *SD* = .79), as was health care services hiring diverse staff (*M* = 4.18, *SD* = 1.23). Participants believed that their cultural background was only slightly to moderately important during their health care visits (*M* = 2.66, *SD* = 1.61). There was a slight preference towards seeing a health care professional from one’s own cultural background (*M* = 3.61, *SD* = 1.33) although participants reported that they rarely were seen by a health care professional from their own background (*M* = 2.06, *SD* = 1.21).

Regarding treatment experiences, reported episodes of racism were very low on average (*M* = 1.39, *SD* = .84). Seventy-seven percent (*n* = 90) of the sample had ‘never’ experienced racism and a further 11% (*n* = 13) had ‘rarely’ experienced racism in health care settings. On average, perceptions of being treated poorly because of one’s cultural background (*M* = 1.72, *SD* = .99) were also generally low. However, 30% (*n* = 35) of the overall sample perceived that they received poor treatment ‘sometimes’ or ‘often’ because of their cultural background. Participants were generally never/rarely afraid to visit western health care services (*M* = 1.40, *SD* = .91). Over 80% (*n* = 94) reported ‘never’ being afraid to access western health care services. Moreover, participants were mostly always/often satisfied with their treatment (*M* = 4.28, *SD* = .90). Less than 3% (*n* = 3) were rarely or never satisfied with treatment received.

#### Cultural sub-groups

Only participants who identified with one of four cultural groups (Latino/a American, Native American, African American/Black, Asian American) were included in the cross-cultural analysis. No significant differences were detected across groups in relation to frequency of health care visits per year χ^2^ [9] = 7.31, *p* = .61. Native American participants (*M* = 40.63, *SD* = 14.16) were significantly older than Latino/a (*M* = 29.55, *SD* = 10.51), African American/Black (*M* = 30.12, *SD* = 11.13) and Asian American (*M* = 25.13, *SD* = 8.78) participant groups, *F*(3,102) = 8.21, *p* < .001. African American/Black (*M* = 3.23, *SD* = 1.11) and Asian American (*M* = 3.40, *SD* = 1.35) participants had on average, obtained slightly higher levels of education compared to Latino/a American and Native American participants, *F*(3,102) = 8.06, *p* < .001. Table 1 presents group differences on the heath service questionnaire by cultural group.

**Table 1:** Health Service Experiences Mean Scores across four Cultural Sub-groups

Item	Total (*N* = 106)	Black/AA (*N* = 26)	Latino(a) (*N* = 33)	Asian (*N* = 15)	Native Am (*N* = 32)	
		M (SD)	M (SD)	M (SD)	M (SD)	F
Are you proud of your cultural heritage?	4.87 (.37)	4.96 (.20)	4.79 (.49)	4.67 (.49)	4.97 (.18)	3.65
Do you feel connected to your culture?	4.42 (.98)	4.58 (.99)	4.24 (.94)	4.20 (1.15)	4.56 (.95)	1.05
How important is your culture to your daily life?	4.26 (1.00)	4.35 (.89)	4.30 (.98)	3.53 (1.19)	4.50 (.88)	3.60
Do you think it is important for healthcare staff to know about different cultures?	2.95 (1.38)	4.85 (.37)^a^	4.55 (.62)^a^	3.87 (1.30)^b^	4.56 (.84)^a^	5.14^*^
Are you afraid to visit Western healthcare services?	1.42 (.94)	1.23 (.59)	1.24 (.71)	1.27 (.70)	1.84 (1.30)	3.27
How important is your culture to your healthcare visits?	2.65 (1.61)	2.50 (1.56)	2.58 (1.64)	1.80 (1.32)	3.25 (1.57)	3.18
Do you think it is important for healthcare services to hire staff from different cultural backgrounds?	4.22 (1.18)	4.46 (1.14)^b^	4.39 (.93)^b^	3.27 (1.53)^a^	4.28 (1.09)^b^	4.26^*^
How often do you see a health care professional from your own cultural background?	2.07 (1.17)	1.96 (1.08)	1.76 (1.03)	2.13 (1.06)	2.44 (1.36)	1.97
Do you feel that you are treated poorly by healthcare professionals because of your cultural background?	1.72 (.99)	1.81 (1.02)	1.42 (.87)^a^	1.27 (.70)^a^	2.16 (1.05)^b^	4.57^*^
Have you experienced racism from your healthcare provider?	1.41 (.86)	1.54 (1.03)	1.22 (.66)	1.07 (.26)	1.66 (1.00)	2.48
Would you prefer to see a health care professional from your own cultural background?	3.68 (1.29)	3.54 (1.30)	3.73 (1.21)	2.93 (1.22)	4.09 (1.28)	3.06
Are you satisfied with the healthcare treatment that you receive?	4.25 (.93)	4.35 (.75)	4.45 (.79)	4.47 (.74)	3.83 (1.18)	3.06
Do you have easy access to health services?	4.58 (1.03)	4.77 (.86)	4.67 (.92)	4.47 (1.25)	4.41 (1.16)	0.73

*N* = 106; *p < .01. Means with different superscripts differ significantly (*p* < .05) within rows. *Black/AA* African American, *Native Am* Native American. Total sample refers to combined total of African American, Latino(a) American, Asian American and Native American participants

All cultural subgroups obtained high mean scores across questionnaire items referring to cultural attachment. While all cultural sub-groups generally agreed that health care services should have staff who know about different cultures and hire diverse staff, Asian participants on average scored significantly lower on these questions compared to the other three cultural groups. Although the importance of culture to health care visits was low to moderate for the overall sample, 50% of Native Americans believed it to be either ‘very’ or ‘extremely’ important.

No significant group differences were detected for the item relating to experiences of racism. The vast majority of participants across cultural groups (Asian American 100%, Latino/a American 94%, Native American 84%, African/American 81%) reported that they had ‘never’ or ‘rarely’ experienced racism from their health care provide. For the item ‘do you feel that you are treated poorly by health professionals because of your cultural background’ 65% of African Americans, 88% of Latino/a Americans, 87% of Asian Americans, and 50% of Native American participants, reported that they had ‘never’ or rarely’ perceived this to be true. Native American participants obtained significantly higher mean scores on this item than Latino/a American and Asian American participants but not African American participants.

No meaningful group differences were observed across items relating to access to services, preference for same-culture professionals and satisfaction with treatment.

### Correlates of health care experiences/outcomes

Table 2 displays the associations between five independent variables (age, level of education, cultural strength, perceptions of poor treatment due to cultural background, experiences of health care provider racism) on a number of health care service related outcomes.

**Table 2:** Predictors of health care attitudes and experiences

	Are you afraid to visit western health care services? (*n* = 115)			Are you satisfied with the treatment you receive? (*n* = 113)			Would you prefer to see a health care professional from your own cultural background? (*n* = 115)		
	B(*SE*)	*t*	*p*	B(*SE*)	*t*	*P*	B(*SE*)	*t*	*p*
Racism	0.32 (.10)	3.16	0.002	−0.20 (.10)	−2.02	0.05	0.17 (.15)	1.13	0.26
Treated Poorly	0.18 (.09)	2.14	0.03	−0.36 (.08)	−4.33	< 0.001	0.26 (.13)	2.04	0.04
Age	0.004 (.01)	0.65	0.52	0.001 (.01)	0.15	0.88	0.001 (.01)	0.08	0.94
Education	−0.18 (.07)	−2.70	0.008	0.13 (.08)	1.67	0.10	−0.15 (.10)	−1.49	0.14
Cultural Strength	−0.01 (.04)	−0.12	0.89	0.12 (.07)	1.83	0.07	0.20 (.06)	3.21	0.002

Racism = ‘Have you experienced racism from your health care provider?’, Treated poorly: ‘Do you feel that you are treated poorly by health professionals because of your cultural background?’

The model predicting ‘are you afraid to visit Western health care services’ was significant *F*(5,110) = 6.83, *p* < .001, *R*^2^ = .24. More frequent experiences of health care provider racism predicted being fearful of western health care services, as did lower levels of education. Being treated poorly because of one’s cultural background was also significantly correlated with being afraid to visit health care services.

The model predicting satisfaction with treatment received by health care services was significant *F* (5,108) = 7.98, *p* < 0.001, *R*^2^ = .27. Being treated poorly because of cultural background was significantly associated with the outcome variable. Here, lower levels of poor treatment were associated with improved satisfaction with health care treatment. The strongest correlate of participant preference for a culturally-matched health service professional was one’s level of personal cultural strength. Being poorly treated because of one’s cultural background also demonstrated a significant relationship with the outcome. The overall model was significant *F*(5,110) = 4.87, *p* < .001, *R*^2^ = .18.

## Discussion

Numerous reports point to the existence of discrimination in health care interactions and inequalities in treatment options and outcomes for particular minority populations [3, 7–9, 24]. This study aimed to identify the self-reported health care experiences and outcomes for a general population of cultural minority Americans in a Midwestern state. Furthermore, and uniquely, the study examined the influence of cultural strength on health care encounters.

### Survey responses

Participants, regardless of cultural background, reported high levels of cultural strength, which was a composite of pride, connection and importance to their lives. Gauging cultural strength is often challenging given that individuals identify or engage with their cultural group(s) in various ways [36]. The three-pronged definition utilised in the study was intended to capture level of identity, the practice of that identity and the significance afforded to that identity. Despite the self-reported high levels of cultural strength in the study, participants generally felt that their culture was less important (or perhaps less relevant) to their health care visits. It is possible that lower scores here reflect participants’ perceptions of the extent to which health care providers consider a minority patient’s culture to be important during health care visits. While no significant mean differences were detected across groups here, a greater proportion of Native American participants did, however, believe that culture was important to their health care visits. This may reflect a preference for holistic traditional healing methods, perhaps as a complementary aspect of conventional care [37].

There appeared to be agreement across cultural groups that health care services should facilitate staff diversity and possess knowledge of different cultures, though Asian-American participants found these proposals to be less important. The diversification of health care staff and attainment of cross-cultural knowledge are recommendations of the cultural competence literature [8]. Participants also showed a slight preference for health care professionals from their own cultural background, supporting previous work [38, 39]. Yet findings from our study also indicate, that despite this inclination appearing on average to be unmet, participants generally still reported strong satisfaction with the health care treatment received. This is not an unusual outcome. A patient’s preference for cultural matching does not necessarily imply that a patient finds it objectionable to receive care from a professional from a different cultural background. However, a cultural matching preference may reflect a perception of in-group comfort [40], particularly for those who may not be proficient in English and/or perceive western health care services to be impersonal and administratively burdensome. In mental health settings, the evidence for the additional benefit of cultural matching on treatment outcomes is meagre [38]. The definition of ‘outcomes’ however, may differ for cultural groups whose conceptualisation of health is more holistic.

Self-reported experiences of health care racism are measured and defined in various ways in the literature, which often impedes precise comparisons in volume. In this study, 88% of the sample had ‘never’ or ‘rarely’ experienced racism in health care settings. Levels of discrimination reported by African Americans and Latino/a Americans in the sample resembled prior research [10, 41]. However, both higher and lower levels of reported racism have been observed elsewhere [42]. Perceived health care racism among Native American participants was largely in correspondence with findings from the available literature [18, 42]. Interestingly, a much larger proportion of participants in the study reported being treated poorly because of their cultural background than those reporting perceived racism. A perception of being treated poorly is a broad concept and can refer to any experience of discrimination during a health care encounter beyond direct racism (i.e., poor communication, being misinformed, not provided with the whole range of treatment options, judgemental staff, feelings of not being respected, needs not met). Prior studies have shown that these experiences are more common among patients of colour compared to white patients [18, 39, 43]. Native American and African American participants reported more frequent experiences of poor treatment compared to Latino/a American and Asian American participants in our study. Both the Native American and African American populations endure pronounced health inequities and are disproportionately represented in lower socio-economic strata, legacies of systemic discrimination, and historical injustices. This may have prompted greater experiences and expectations of poor treatment. Specifically, half of the Native American participants reported that they had received poor treatment ‘sometimes’ or ‘often’. Native Americans have reported higher levels of health care discrimination compared to other minority groups in prior research [18, 44]. Discrimination towards First Nations peoples in health care settings worldwide has been documented [45]. There are a number of potential reasons that underlie the higher rates of poor treatment perceived by Native American participants. Due to history of colonisation, marginalisation and exclusion, Native Americans may be subject to more frequent mistreatment than other minority groups in the U.S. Another contributor may be the fact that the Indian Health Service, which provides health service delivery for members of recognised tribes, has been described as underfunded, inaccessible to some, and offering a limited range of services [46–48]. Moreover, Native Americans who subscribe to more traditional-oriented health models and healing practices may perceive conventional health services as unable to meet their needs. Historical marginalization has also perhaps prompted a degree of mistrust for social institutions among some Native Americans. This is a phenomenon that has been noted in other Indigenous populations [24, 25]. Similarly, in a Canadian survey of more than 2000 First Nations people, 18% reported negative experiences with health care services [49]. The main reason underpinning negative experiences with health care services was reported to be ‘poor treatment’ [49]. Research has also illustrated that some Native Americans experience anticipatory discrimination, whereby there is an expectation of poor treatment or a lesser standard of care because of one’s Native American and socio-economic status [50, 51]. Despite participants on average being generally satisfied with the treatment they were receiving, the proportion of Native Americans and African Americans who reported some degree of perceived poor treatment is concerning.

#### Correlates of health experiences/outcomes

Identifying the predictors of three important health care experiences and outcomes provided some insight into patient behaviours. Past experiences of perceived racism were significantly related to a fear of conventional health services. However, previous racism was not meaningfully correlated with treatment satisfaction nor a preference for cultural-matching. Perceptions of poor treatment were instead significantly associated with treatment satisfaction, reflecting prior research [10]. Moreover, cultural strength was strongly associated with a preference for cultural-matching.

Past experiences of racism in health care settings perhaps generates a level of apprehension regarding future appointments. However, clinically poorer treatment is in fact related to diminished treatment satisfaction. This finding is unsurprising - the perception that a health service is providing an individual with a lesser (or restrictive) service is likely to engender lower levels of satisfaction. The finding that past racism was less connected to treatment satisfaction may suggest that participants viewed perceived episodes of racism during a health care visit as detached or coincidental to the actual medical treatment they were receiving. Racist incidents may be viewed as a frustrating occurrence while the inability of the health service to meet one’s health needs (or culturally specific health needs) is perhaps viewed as more problematic. Furthermore, past racism was not associated with a preference for a culturally similar practitioner. Rather, a participant’s cultural strength proved to be the strongest predictor here. A strong attachment to culture as defined in this study infers an everyday significance, or perhaps a ‘way of life’. Individuals with this level of cultural attachment may prefer a culturally-matched clinician, the assumption being that this clinician is part of the in-group and likely to be familiar with unique cultural considerations and needs [39]. Prior research has found a strong association between cultural affiliation and the use of traditional health practices [37]. Aside from the relationship between cultural strength and preference for cultural matching, cultural strength was not associated with treatment satisfaction nor fear of conventional health settings in this study.

Another finding of note was the relationship between education and fear of services. Possessing a lower level of education predicted a greater fear of conventional services. Less educated participants may have lower levels of health literacy [52–54] and perhaps feel judged or intimidated by health service processes. In addition, people with low educational attainment may be unaware of or avoid health services and only come into contact with them during emergency situations. This may confirm an already held belief that health services are frightening and associated with serious medical interventions and/or death. Moreover, a large survey of First Nations in Canada discovered that those with lower levels of education were more likely than educated participants to perceive non-Indigenous services to be ineffectual [49]. Furthermore, older age is associated with lower educational attainment, particularly in Indigenous populations [55]. In this study, Native American participants were significantly older than participants from the other cultural groups. In the United States, Native Americans have lower graduation rates compared to other cultural groups, and those who are older tend to have lower educational attainment [56]. This may have contributed to the relationship between education and fear of health services. Furthermore, health professionals often speak a different ‘language’ comprising medical jargon which can make communication with some patients more challenging and less accessible. This can promote shame and lack of questioning by some patients who may not question their treatment as a result [57].

Lower levels of education. However, were not meaningfully associated with treatment satisfaction nor a preference for cultural matching.

### Implications and limitations

The bulk of the sample reported few if any, experiences of racism in health care settings. However, participants who had experienced racism were more likely to be afraid of visiting typical health care services. Further research is required to unpack the nature of the perceived racism experienced so that service providers can identify and actively minimise these episodes. It may also be necessary to hire multicultural community health liaison officers to provide assurance and assistance to minority individuals who are fearful of attending health services. The notable minority of Native Americans and African Americans who reported poor treatment is of some concern. First Nations peoples worldwide experience this discrimination. These types of poor treatment often have a historical and political significance that has failed to be addressed. Again, further research is necessary to identify where exactly during the health care experience patients believed they were being treated poorly or differently. It is also important to identify whether perceived discrimination is a result of genuine prejudice (explicit or implicit) on behalf of the service. For example, a service with restricted options for treatment or unhelpful staff may be reflective of underfunding. Similarly, general ineptitude may be confused for cultural bias. Either way, the hiring of staff from diverse backgrounds may alter the attributions made by minority patients for poor interpersonal experiences, alleviating feelings of being judged or misunderstood for some minority patients. Indeed, patients who are culturally strong appear to preference health care professionals from their own cultural background. Although the evidence for culture matching on treatment outcomes is equivocal, it may be that cultural matching affords a patient with a level of comfort or security during the clinical interaction. Cross-cultural training for staff may be another consideration. Again, the evidence for such training on patient outcomes is weak [58, 59]. The focus of training could instead be on the social determinants of health for racial and cultural minorities and how health professionals can better recognise barriers to access and treatment adherence [60]. A stronger commitment to patient-centred care which aims to provide health services that reflect the needs of the patient and not the mainstream health system, is recommended.

The study has a number of limitations. Sampling was non-representative, although undertaken at popular, central public place (shopping mall) and a well-attended cultural event. The study may have also over-selected for individuals who were confident, educated, literate and who possessed a higher English language proficiency. The availability of bi-lingual researchers and the option to have the survey read to participants may have alleviated some of these concerns. Several interviews were carried out in Spanish. The study captured the bulk of minority individuals who were in proximity to our research stalls. The sample sizes are small by cultural group because such groups are underrepresented in the particular region the study was held. This precluded identifying correlates of health care experiences by cultural group. Also, the definitions of racism and being ‘treated poorly’ were broad and in some ways left to the discretion of the participant. While this prevented the identification of explicit incidents, it allowed participants to consider any incident they perceived to be discriminatory or substandard. Similarly, the components of cultural strength were determined by participant perception as opposed to a more objective or detailed measure of cultural attachment (i.e., how often do you participate in cultural activities; which cultural activities do you participate in; Knowledge of traditional customs/languages). This may have inflated the cultural strength of some participants, although cultural attachment/engagement is very much a personal matter and people identify with their cultural group in many different ways. The intent of the study was to gather preliminary information on health care experiences via a succession of relevant questions. However further research exploring the psychometric properties of the questionnaire at large may be warranted. Specific items may need to be refined. Open-ended questions may need to be included to allow for greater context. This process may benefit from direct consultation with minority and underserved communities. Finally, personal/family income levels and the health insurance status of the participants were not obtained. Several participants may have accessed health services through Medicaid, a local public health center or the Indian Health Service which may have influenced their experiences. Nonetheless, participants on average reported adequate access to services. The study had a number of strengths. It provided a rare insight into minority health care expectations and experiences in a region with comparatively lower proportions of racial and cultural minorities. Additionally, the study explored the impact of cultural strength on health care interactions and outcomes.

## Conclusion

In this study, participants on average, reported that they had good access to health care services, were not afraid to visit conventional services, experienced low levels of racism and poor treatment and were generally satisfied with their treatment. Participants also reported high levels of cultural strength, and viewed staff diversity and staff knowledge of different cultures as important. The importance of culture to health care visits was somewhat important as was a preference for practitioner cultural matching. Native American participants reported higher rates of poor treatment and believed culture to be more important to their health care visits compared to other cultural groups. Experiences of health care provider racism and lower levels of education predicted being afraid of attending conventional health care services. Being treated poorly because of one’s cultural background in healthcare settings predicted satisfaction with treatment. And finally, cultural strength, and poor treatment predicted a preference for cultural matching.
